# Respiratory- and cardiac-triggered three-dimensional sheath inked rapid acquisition with refocused echoes imaging (SHINKEI) of the abdomen for magnetic resonance neurography of the celiac plexus

**DOI:** 10.1186/s41747-019-0095-4

**Published:** 2019-03-29

**Authors:** Cyril J. Ferrer, Clemens Bos, Masami Yoneyama, Makoto Obara, Lisanne Kok, Maarten S. van Leeuwen, Ronald L. A. W. Bleys, Chrit T. W. Moonen, Lambertus W. Bartels

**Affiliations:** 1 Imaging Division, University Medical Center Utrecht, Utrecht University, Heidelberglaan 100, 3584 CX Utrecht, The Netherlands; 2 Philips Japan, 3-37 Kohnan 2-chome, Minato-ku, Tokyo, 108-8507 Japan; 3 Department of Radiology, University Medical Centre Utrecht, Utrecht University, Heidelberglaan 100, 3584 CX Utrecht, The Netherlands; 4 Department of Anatomy, University Medical Centre Utrecht, Utrecht University, Heidelberglaan 100, 3584 CX Utrecht, The Netherlands; 5 Utrecht University, Domplein 29, 3512 JE Utrecht, The Netherlands

**Keywords:** Abdomen, Celiac plexus, Magnetic resonance imaging, Organ motion

## Abstract

The visualisation of the celiac plexus using respiratory- and cardiac-triggered three-dimensional (3D) sheath inked rapid acquisition with refocused echoes imaging (SHINKEI) was evaluated. After ethical approval and written informed consent, eight volunteers (age 27 ± 5 years, mean ± standard deviation) were scanned at 1.5 and 3 T. Displacement of the celiac ganglia due to aortic pulsatility was studied on axial single-slice breath-hold balanced turbo field-echo cine sequences in five volunteers and found to be 3.0 ± 0.5 mm (left) and 3.1 ± 0.4 mm (right). Respiratory- and cardiac-triggered 3D SHINKEI images were compared to respiratory- and cardiac-triggered fat-suppressed 3D T2-weighted turbo spin-echo and respiratory-triggered 3D SHINKEI in all volunteers. Visibility of the celiac ganglia was rated by three radiologists as visible or non-visible. On 3D SHINKEI with double-triggering at 1.5 T, the left and right ganglia were seen by all observers in 7/8 and 8/8 volunteers, respectively. At 3 T, this was the case for 6/8 and 7/8 volunteers, respectively. The nerve-to-muscle signal ratio increased from 1.9 ± 0.5 on fat-suppressed 3D T2-weighted turbo spin-echo to 4.7 ± 0.8 with 3D SHINKEI. Anatomical validation was performed in a human cadaver. An expert in anatomy confirmed that the hyperintense structure visible on *ex vivo* 3D SHINKEI scans was the celiac plexus. In conclusion, double-triggering allowed visualisation of the celiac plexus using 3D SHINKEI at both 1.5 T and 3 T.

## Key points

- Over the cardiac cycle, the left and right celiac ganglia showed a displacement of respectively 3.0 ± 0.5 and 3.1 ± 0.4 mm, which can be compensated on three-dimensional (3D) sheath inked rapid acquisition with refocused echoes imaging (SHINKEI) by applying cardiac triggering.
- In the abdomen, respiratory- and cardiac-triggered 3D SHINKEI showed superior nerve-to-muscle signal ratios as compared to respiratory-triggered 3D SHINKEI.
- Respiratory- and cardiac-triggered 3DSHINKEI allowed a high-resolution visualisation of the celiac plexus.

## Background

Magnetic resonance neurography (MRN) methods allow visualisation of peripheral nerves by generating contrasts based on the relatively long T2 relaxation time of endoneurial fluid [1] and/or based on restricted diffusion within axons [2, 3]. Recent peripheral nerve imaging studies have demonstrated a potential for depiction of three-dimensional (3D) nerve anatomy and disorders using MRN techniques such as T2-weighted turbo spin-echo (TSE) and diffusion-weighted imaging (DWI) in both the lumbosacral and the brachial plexus [2, 4]. Advantages and limitations have been shown for either approach: exploiting the contrasts based on differences in T2 delivers high-resolution images which typically show nerve signal isointense to that of slowly flowing blood. On DWI, nerves are hyperintense, but since DWI commonly employs echo-planar imaging (EPI) read-outs, it often suffers from relatively low spatial resolution and image distortion [5, 6].

Recently, 3D sheath inked rapid acquisition with refocused echoes imaging (SHINKEI) was introduced, which combines an improved motion-sensitised driven equilibrium (iMSDE) prepulse with a T2-weighted fat-suppressed sequence in order to suppress signal of slow flowing blood [7, 8]. In a recent study, 3D SHINKEI of the lumbosacral plexus was shown to increase the contrast of nerve to the surrounding tissue as compared to 3D inversion-recovery TSE, resulting in better conspicuity of smaller nerve branches [9].

In the abdomen, MRN methods have not yet been implemented. These methods would have value for imaging the celiac plexus, a dense neuronal network in the retroperitoneum which surrounds the celiac trunk and the root of the superior mesenteric artery (SMA) and includes two large celiac ganglia [10]. However, the complex anatomy and the organ motion due to respiration and peristalsis pose challenges for MRN in the abdomen. Moreover, the celiac plexus might also be subject to tissue displacement caused by pulsation of the aorta [11]. We hypothesised that 3D SHINKEI is a good candidate for imaging the celiac plexus, based on its capabilities to suppress signal from background tissues and from slowly flowing blood, provided that cardiac and respiratory motion are adequately compensated.

In this study, we evaluated whether double-triggered 3D SHINKEI improved visualisation of the celiac plexus as compared to the visualisation obtained with respiratory-triggered 3D SHINKEI and double-triggered fat-suppressed 3D T2-weighted TSE. Because of the known location of the celiac plexus close to the aorta, tissue displacement was expected in the target area. Therefore, we characterised the motion over the cardiac cycle. In addition, an anatomical validation was performed in a human cadaver.

## Methods

Ethical approval for the volunteer study aimed at developing a magnetic resonance imaging (MRI) protocol had been granted by the medical ethics committee of the University Medical Center of Utrecht (NL53099.041.15), and written informed consent was obtained from all volunteers. Eight healthy volunteers (age 27 ± 5 years, mean ± standard deviation) were scanned in two independent sessions at 1.5 and 3 T using clinical scanners (Ingenia, Philips Healthcare, Best, The Netherlands). Volunteers were scanned in supine position. Images were acquired using a 16-element array anterior coil and a 12-element array posterior coil for signal reception.

### Aortic pulsatility imaging and displacement measurements

An axial single-slice breath-hold balanced turbo field-echo cine sequence was acquired two times in the same scan session at the level of the SMA in five volunteers. The mixed image contrast of this sequence provides high fluid signal and background tissue contrast, making it attractive for measuring the tissue displacement caused by aortic pulsatility at the location of the celiac ganglia. Data were acquired over multiple heartbeats using vector cardiogram, triggering and retrospectively binned into 30 heart phases. Scan parameters were repetition time 2.9 ms, echo time 1.44 ms, turbo factor 17, reconstructed voxel size 0.99 × 0.99 × 8 mm^3^, scan duration 9 s, and number of excitation 1. An optical flow-based method [12] was used to register the cine images so as to obtain displacement maps for all cardiac phases. These maps were used to determine tissue displacement inside circular regions of interest positioned at the expected locations of the celiac ganglia. Next, the cardiac trigger delay was set to be as short as possible to allow MRN data acquisition during the most favourable part of the cardiac cycle.

### *In vivo* MRN of the celiac ganglia

Fat-suppressed 3D T2-weighted TSE and 3D SHINKEI data was acquired with the parameter settings summarised in Table 1. All volunteers were scanned in two independent sessions at 1.5 and 3 T using the following protocol: (1) respiratory- and cardiac-triggered fat-suppressed 3D T2-weighted TSE, (2) respiratory-triggered 3D SHINKEI, and (3) respiratory- and cardiac-triggered 3D SHINKEI.

**Table 1:** Scan parameter settings of 3D T2-weighted TSE and 3D SHINKEI sequences

Sequence/parameter	Field of view (mm)	Resolution (mm^3^)	TR/TE (ms)	Flip angle (degrees)	Bandwith per pixel (Hz)	iMSDE
3D T2-weighted TSE	250 × 250 × 100	1.1 × 1.1 × 1.0	3000/57	90	431.7	Not applied
3D SHINKEI (1.5 T)	250 × 250 × 100	1.25 × 1.25 × 2.5	2400/51	90	1034.3	*V*_enc_ = 1 cm/s *T*_prep_ = 50 ms
3D SHINKEI (3 T)	250 × 250 × 100	1.0 × 1.0 × 2.0	2400/61	90	1034.3	*V*_enc_ = 1 cm/s *T*_prep_ = 50 ms

*3D* three-dimensional, *iMSDE* improved motion-sensitised driven equilibrium, *SHINKEI* sheath inked rapid acquisition with refocused echoes imaging, *TE* echo time, *T*_prep_ iMSDE preparation duration, *TR* repetition time, *TSE* turbo spin-echo, *V*_enc_ velocity encoding

Physiology signals for triggering were obtained using a respiratory belt and a peripheral pulse unit. The respiratory trigger delay was set to 400 ms, such that the acquisition window would start in a stable expiration phase. In all acquisitions, three saturation bands were added to further suppress peristaltic motion artifacts.

The visibility of the celiac ganglia was assessed by three trained radiologists with respectively 4, 11, and 4 years of experience (Table 2). Visibility was rated on a binary scale: 0 if not visible and 1 if visible. When visible, the ganglia were manually segmented slice by slice by a single observer (C.J.F.) and labeled as the left or right ganglia depending on their position relative to the main vascular landmarks (aorta, SMA, and celiac trunk). The number of volunteers in whom ganglia were visible was reported for every sequence at both field strengths and for the left and right ganglia separately. Ganglia were considered visible if all three readers could independently identify the structure.

**Table 2:** Visibility of the left and right ganglion per sequence and field strength. 2T refers to the double respiratory and cardiac triggering

	Resp 3D-SHINKEI		2T 3D-SHINKEI		2T 3D-T2w TSE	
1.5 T	Left	Right	Left	Right	Left	Right
3 observers	0	1	7	8	6	7
2 observers	2	1	0	0	1	1
1 observer	0	0	1	0	1	0
0 observer	6	6	0	0	0	0
	Resp 3D-SHINKEI		2T 3D-SHINKEI		2T 3D-T2w TSE	
3 T	Left	Right	Left	Right	Left	Right
3 observers	1	0	6	8	6	8
2 observers	0	4	1	0	2	0
1 observer	1	1	1	0	0	0
0 observer	6	3	0	0	0	0

The nerve signal *S*_*n*_ in the regions of interest of the ganglia was compared to the signal reference *S*_*m*_ of the musculus erector spinae, by calculating the signal ratio as follows:

$$ {R}_{nm}=\frac{S_n}{S_m} $$

### *Ex vivo* anatomical validation study

A formaldehyde-fixated human cadaver torso was derived from a body, which entered the department of anatomy through a donation program. Written informed consent was obtained during life that allowed using the entire body for educational and research purposes. The torso was scanned at room temperature in supine position on a 3-T scanner (Ingenia, Philips Healthcare, The Netherlands), using the same protocols and parameter settings as those for volunteer scanning, except for a fixed repetition time of 4000 ms. After MRI, a 15-cm^3^ frozen volume containing the celiac plexus region was cut from the torso. Axial slices were cut using a cryomacrotome (CM3600 XP, Leica, Wetzlar, Germany) and photographed at 0.5-mm intervals, with a field of view of 200 × 150 mm^2^ with a matrix of 2560 × 1920. The obtained cryomacrotome images were used as the reference *ex vivo* data set.

This reference *ex vivo* data set was co-registered to the cadaver images. First, a manual initialisation with rigid transformation was done. Secondly, an affine registration was performed using in-house developed software (Elastix, Image Science Institute, Utrecht, The Netherlands) [13]. An expert in anatomy with over 25 years of experience (R.L.A.W.B.) was asked to identify the celiac ganglia on the reference *ex vivo* images. Then, image overlay was used to assess the correspondence between the structures identified as the celiac ganglia on the reference *ex vivo* images and *ex vivo* MRI, respectively.

### Statistical analysis

Tissue displacement and signal ratio, *R*_*nm*_, were reported as median and range over all subjects. *R*_*nm*_ distribution values were compared using a Wilcoxon signed-rank test: comparisons were performed per ganglion and between double-triggered 3D SHINKEI and 3D T2-weighted TSE. A *p* value lower than 0.05 was considered statistically significant.

## Results

### Aortic pulsatility imaging and displacement measurements

**Fig. 1:**
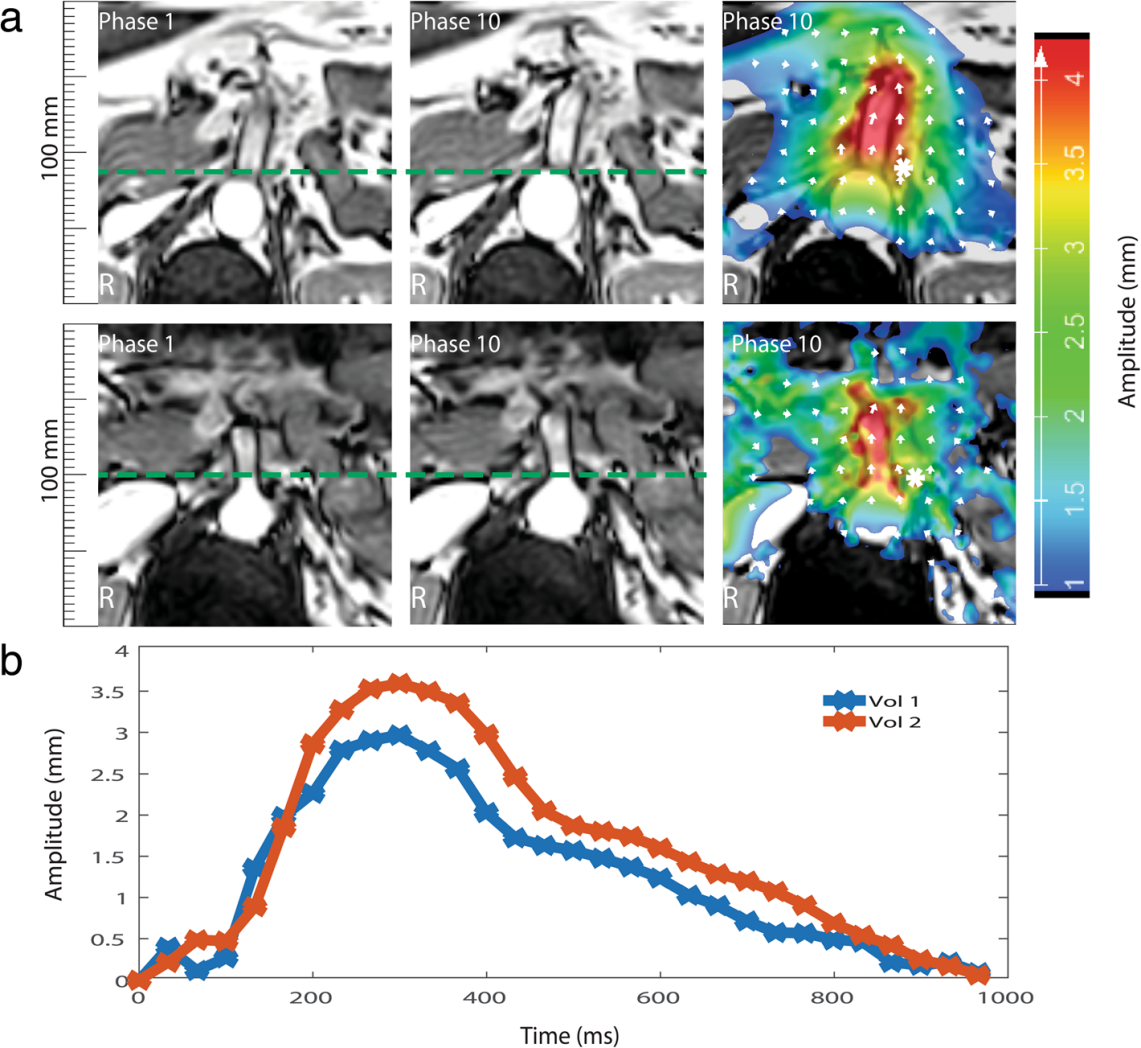
**a** Representative cine images acquired in two volunteers at the level of the superior mesenteric artery. Phases with minimum (left) and maximum (middle) aorta diameter are shown, *i.e.*, phase 1 of 30 and 10 of 30, respectively. The induced displacement amplitude over the cardiac cycle is presented as a colour coded overlay on the anatomy (right). Arrows illustrate amplitude and direction of the displacements. **b** Variation of the motion amplitude over the cardiac cycle. White asterisks in **a** indicate where the displacement was evaluated

Two representative examples of cine images at minimum and maximum cross-sectional area of the aorta over the heart cycle illustrate the induced tissue displacements seen at the level of the SMA (Fig. 1). The area of the plexus moved mainly in the anteroposterior direction, with maximum displacements of approximately 3 to 3.5 mm immediately near the aorta where the celiac plexus was expected to be located (see Fig. 1a, b). Displacements decreased with distance from the aorta, but remained well over 1 mm within a 50-mm diameter area (see Fig. 1a). The subjects had a relatively stationary phase of approximately 600-ms duration, during which the displacements were less than 1.5 mm (see Fig. 1b). Similar results were obtained in all five volunteers. On average, the left and right ganglia displacements were found to be 3.0 mm (range 2.4–3.6 mm) and 3.1 mm (range 2.5–3.9 mm), respectively.

### *In vivo* MRN of the celiac ganglia

**Fig. 2:**
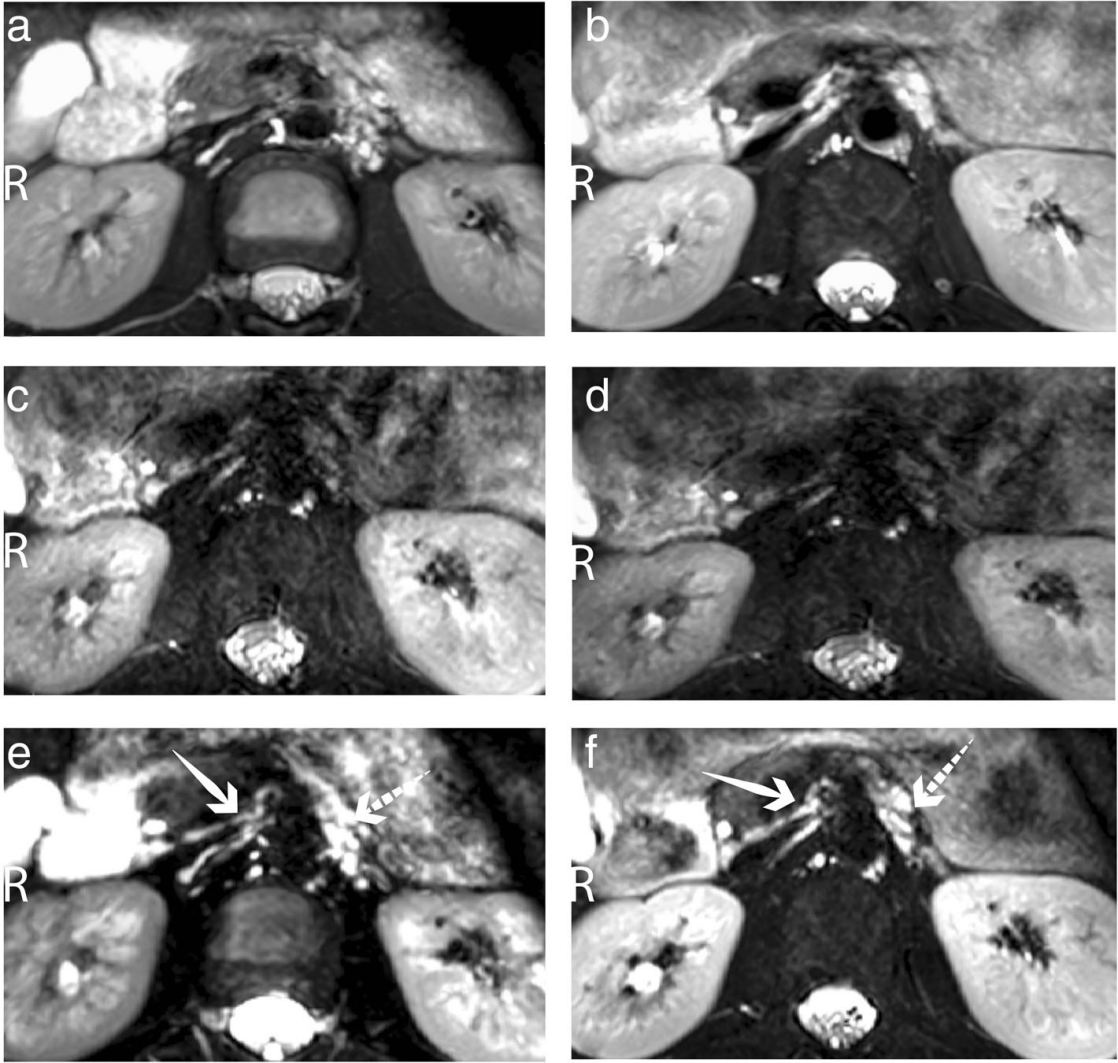
Typical axial-acquired image, centred at the level of the celiac trunk, obtained with 3D T2-weighted TSE and 3D SHINKEI sequences in the healthy volunteer #1. 3D SHINKEI images with respiratory triggering only at 1.5 T (**c**) and 3 T (**d**) show significant motion artifacts: blurring, signal loss, and noise band in phase direction. These artifacts mostly disappeared after adding cardiac synchronisation to the 3D T2-weighted TSE sequence at 1.5 T (**a**) and 3 T (**b**) or to the 3D-SHINKEI sequence at 1.5 (**e**) and 3 T (**f**). The right (solid white arrows) and left ganglia of the celiac plexus (dashed white arrow) are clearly distinguishable as hyperintense signals

A representative case of the improved image quality obtained using double-triggering at the level of the celiac plexus is shown in Fig. 2. In this example, the ganglia present as a laminar-shaped hyperintense structure on the right side of the celiac trunk and multiple hyperintense structures on the left side. On respiratory-triggered 3D SHINKEI images, only in a single volunteer a ganglion was identified by all observers: a left ganglion at 3 T and a right ganglion at 1.5 T. As shown in Table 2, with double-triggered 3DSHINKEI and 3D T2-weighted TSE images, at 3 T, eight out of eight right ganglia and six out of eight left ganglia were visible; at 1.5 T, on double-triggered 3D-SHINKEI images, eight out of eight right ganglia and seven out of eight left ganglia were visible, while on double-triggered fat-suppressed 3D T2-weighted TSE images, the right ganglion was visible in seven out of eight and the left ganglion in six out of eight volunteers.

**Fig. 3:**
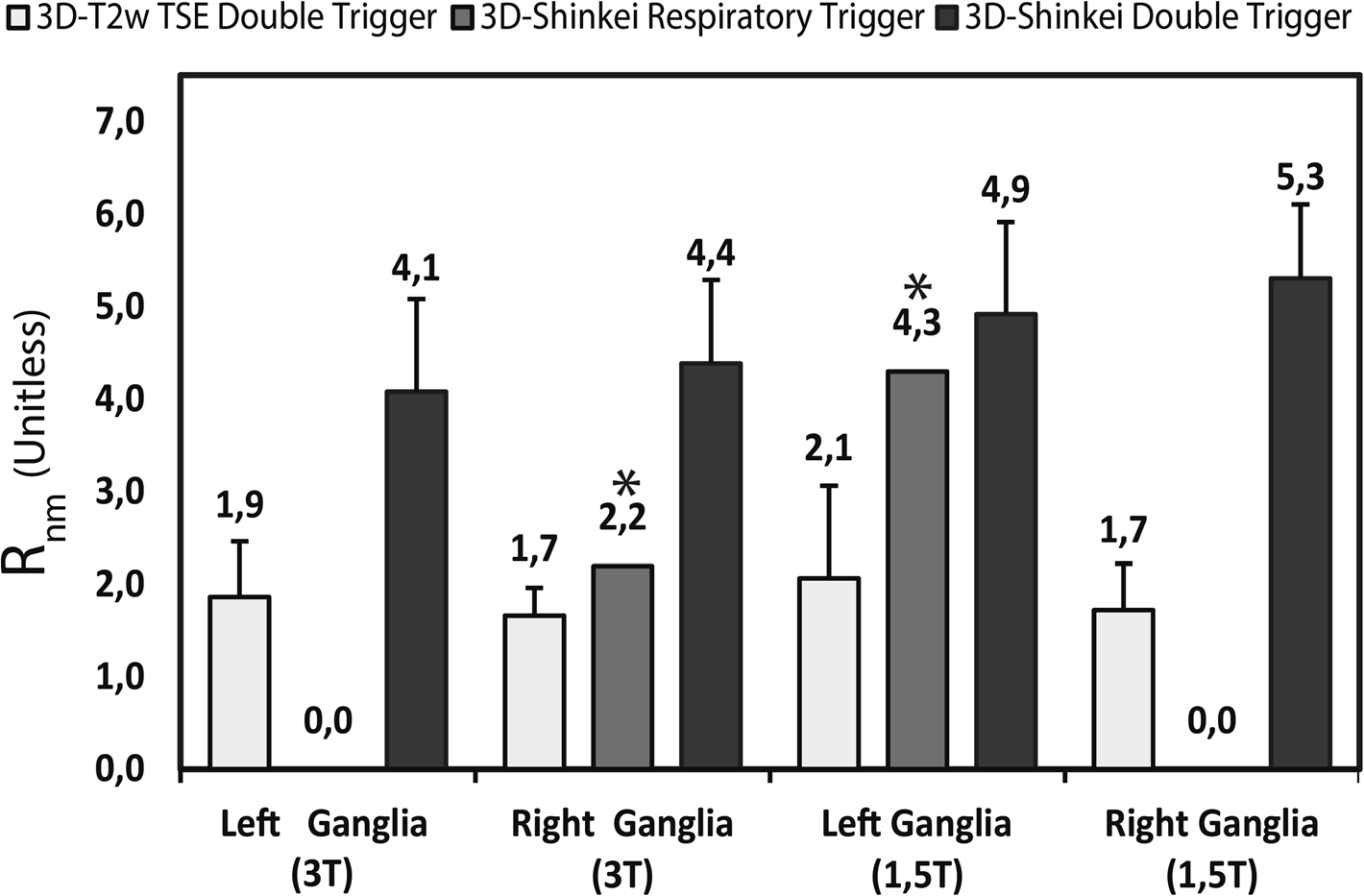
Nerve-to-muscle signal ratio (*R*_*nm*_) at 3 T and 1.5 T for the 3D T2-weighted TSE, the respiratory-triggered 3D SHINKEI, and the double motion-compensated 3D SHINKEI sequences. The asterisk indicates that the measurement could only be done in one volunteer

Quantitative analysis showed that nerve-to-muscle signal ratio was consistently higher on the double-triggered 3D SHINKEI images compared to fat-suppressed 3D T2-weighted TSE images (Fig. 3). At 3 T, the 3D-SHINKEI signal ratios were 4.2 (range 3.4–6.1) and 3.7 range (3.0–5.8) for the right and left ganglia, respectively, with a significant increase (*p* < 0.001) with respect to the signal ratios on fat-suppressed 3D T2-weighted TSE of 1.6 (range 1.3–2.2), right ganglia, and 1.7 (range 1.2–3.0), left ganglia. Likewise, the signal ratios at 1.5 T increased from 1.7 (range 1.1–3.0), right ganglia and 1.6 (range 1.2–4.1), left ganglia on fat-suppressed 3D T2-weighted TSE images to 5.7 (range 3.8–6.0) and 5.0 (range 3.3–6.5) on 3D SHINKEI images, respectively (*p* < 0.001).

### *Ex vivo* anatomical validation study

**Fig. 4:**
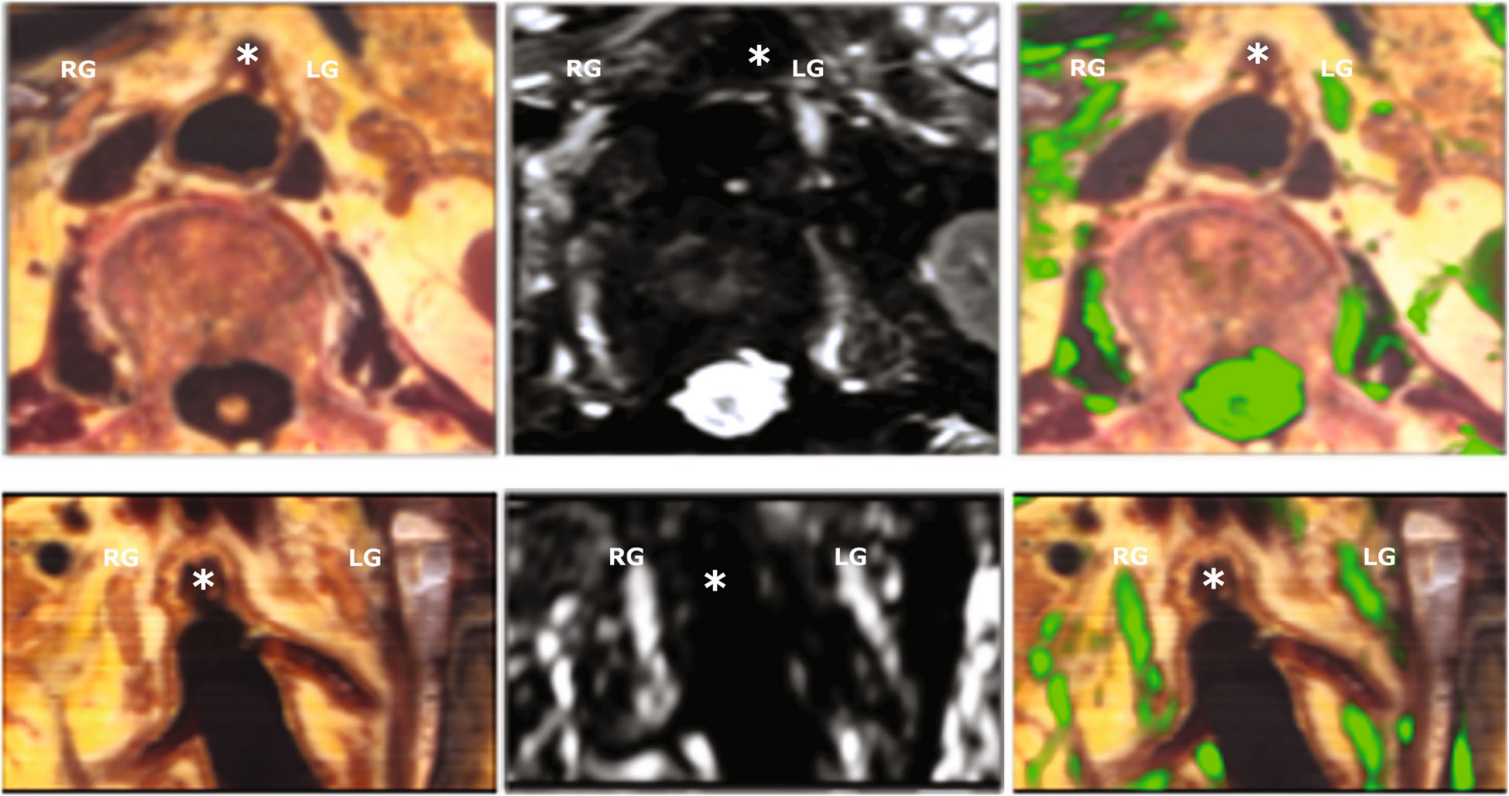
Comparison of *ex vivo* cryomacrotome images and 3D SHINKEI images. The first row shows axial slices at the level of the superior mesenteric artery trunk: the cryomacrotome section (left), the corresponding 3D SHINKEI (middle), and an overlay (in green) of the 3D SHINKEI image on the corresponding cryomacrotome image (right). The second row shows the same data set reformatted in the coronal plane. The white asterisk indicates the position of the superior mesenteric artery. The locations of the right and left ganglia of the celiac plexus are indicated by RG and LG, respectively

Based on colour, location, configuration, size, and tissue contrast, the expert in anatomy identified two structures on the *ex vivo* reference image as the left and right celiac ganglia (Fig. 4). These locations coincided with that of distinctly hyperintense structures on 3D SHINKEI images (see Fig. 4). In a sagittal reformat, the two ganglia can be identified as elongated structures following the aorta from the level of the SMA to the level of the renal arteries (see Fig. 4). In terms of location and shape, the appearance of the celiac plexus on the *ex vivo* MRI corresponded to that on the *in vivo* MRI.

## Discussion

In this study, we showed the feasibility of imaging the celiac plexus in volunteers by using a 3D SHINKEI sequence with combined respiratory and cardiac triggering. This motion compensation scheme substantially improved the visibility of structures identifiable as the left and right ganglia with respect to respiratory triggering only. The double-triggered 3D SHINKEI sequence also increased the signal to background ratio as compared to double-triggered fat-suppressed 3D T2-weighted TSE. Comparison of the location, shape, and aspect of the celiac ganglia in the *ex vivo* reference and MRI sets from the cadaver study supported the anatomical interpretation of the *in vivo* volunteer scans. As such, double-triggered 3D-SHINKEI holds the potential for further evaluation in clinical and research settings.

In this study, we measured the motion of the celiac plexus caused by aortic distensibility. In our population, the measured displacements were in a range of 3 to 3.5 mm. From the literature, it is known that the size of the ganglia vary between 5 and 45 mm [14, 15]. The observed motion amplitude therefore amounts to 10 to 75% of the size of the structures of interest. This observation is a likely explanation for the superior conspicuity of the celiac ganglia on double motion-compensated imaging strategies as compared to methods using respiratory triggering only. Similar improvement of abdominal imaging with combined respiratory and cardiac triggering has also been demonstrated in the context of diffusion-weighted imaging [16, 17], albeit that the mechanism for the sensitivity to the cardiac cycle in that case is mostly understood in terms of tissue deformation, rather than as being caused by displacement of small anatomical structures.

High-resolution imaging of these structures would be of potential interest in different contexts. The celiac plexus is involved in various pain transfer mechanisms and is a target for image-guided pain palliation intervention [18]. The efficacy of these procedures relies on precise targeting of the celiac plexus [19], which shows considerable anatomical variability [20]. In addition, improved neurographic image quality in this anatomical region could also be of added value for tumour staging [21, 22].

For this feasibility study, the evaluation was limited to assessing the visibility and contrast of the celiac ganglia. Further investigations are necessary to evaluate the value of the proposed method in radiological assessments. Also, this study was conducted in young healthy volunteers, in whom the selected triggering scheme led to a steady acquisition pattern. Further studies are required to evaluate the performance of double-triggering in patients, who have a higher risk of uncontrolled motion that might lead to the deterioration of the image quality. In addition, aortic distensibility is known to decrease with age, which may influence the necessity for cardiac compensation in a typical patient population [23].

In conclusion, double-triggering allows visualisation of the celiac plexus using fat-suppressed 3D T2-weighted TSE and 3D SHINKEI at both 1.5 and 3 T. Contrast of the celiac ganglia was highest on 3D SHINKEI images.

## Abbreviations

3D: Three-dimensional
DWI: Diffusion-weighted imaging
EPI: Echo-planar imaging
iMSDE: Improved motion-sensitised driven equilibrium
MRI: Magnetic resonance imaging
MRN: Magnetic resonance neurography
SHINKEI: Sheath inked rapid acquisition with refocused echoes imaging
SMA: Superior mesenteric artery
TSE: Turbo spin-echo

## References

[1] Chhabra A, Andreisek G, Soldatos T (2011). MR neurography: past, present, and future. AJR Am J Roentgenol.

[2] Eppenberger P, Andreisek G, Chhabra A (2014). Magnetic resonance neurography. Diffusion tensor imaging and future directions. Neuroimaging Clin N Am.

[3] Ahlawat S, Chhabra A, Blakely J (2014). Magnetic resonance neurography of peripheral nerve tumors and tumorlike conditions. Neuroimaging Clin N Am.

[4] Soldatos T, Andreisek G, Thawait GK (2013). High-resolution 3-T MR neurography of the lumbosacral plexus. Radiographics.

[5] Koyasu S, Iima M, Umeoka S (2014). The clinical utility of reduced-distortion readout-segmented echo-planar imaging in the head and neck region: initial experience. Eur Radiol.

[6] Stocker D, Manoliu A, Becker AS (2018). Image quality and geometric distortion of modern diffusion-weighted imaging sequences in magnetic resonance imaging of the prostate. Invest Radiol.

[7] Wang J, Yarnykh VL, Yuan C (2010). Enhanced image quality in black-blood MRI using the improved motion-sensitized driven-equilibrium (iMSDE) sequence. J Magn Reson Imaging.

[8] Yoneyama M, Takahara T, Kwee TC, Nakamura M, Tabuchi T (2013) Rapid high resolution MR neurography with a diffusion-weighted pre-pulse. Magn Reson Med Sci 12:111–119 10.2463/mrms.2012-0063 23666153

[9] Hiwatashi A, Togao O, Yamashita K (2017). Evaluation of chronic inflammatory demyelinating polyneuropathy: 3D nerve-sheath signal increased with inked rest-tissue rapid acquisition of relaxation enhancement imaging (3D SHINKEI). Eur Radiol.

[10] Mitchell GAG (1953) Anatomy of the autonomic nervous system. Edinburgh, E. & S. Livingstone

[11] Van Prehn J, Vincken KL, Sprinkhuizen SM (2009). Aortic pulsatile distention in young healthy volunteers is asymmetric: analysis with ECG-gated MRI. Eur J Vasc Endovasc Surg.

[12] Zachiu C, Papadakis N, Ries M, Moonen C, Denis de Senneville B (2015) An improved optical flow tracking technique for real-time MR-guided beam therapies in moving organs. Phys Med Biol 60:9003–9029 10.1088/0031-9155/60/23/9003 26540256

[13] Klein S, Staring M, Murphy K, Viergever MA, Pluim JP (2010) Elastix: a toolbox for intensity-based medical image registration. IEEE Trans Med Imaging 29:196–205. 10.1109/TMI.2009.2035616 10.1109/TMI.2009.2035616 19923044

[14] Zhang XM, Zhao QH, Zeng NL et al (2006) The celiac ganglia: anatomic study using MRI in cadavers. AJR Am J Roentgenol 186:1520–1523 10.2214/AJR.04.1765 16714639

[15] Ward EM, Rorie DK, Nauss LA, Bahn RC (1979). The celiac ganglia in man: normal anatomic variations. Anesth Analg.

[16] Mürtz P, Flacke S, Träber F, van den Brink JS, Gieseke J, Schild HH (2002) Abdomen: diffusion-weighted MR imaging with pulse-triggered single-shot sequences. Radiology 224:258–264 10.1148/radiol.2241011117 12091693

[17] Mürtz P, Krautmacher C, Trüber F, Gieseke J, Schild HH, Willinek WA (2007) Diffusion-weighted whole-body MR imaging with background body signal suppression: a feasibility study at 3.0 Tesla. Eur Radiol 17:3031–3037 10.1007/s00330-007-0717-8 17646990

[18] Loukas M, Klaassen Z, Merbs W, Tubbs RS, Gielecki J, Zurada A (2010) A review of the thoracic splanchnic nerves and celiac ganglia. Clin Anat 23:512–522 10.1002/ca.20964 20235178

[19] Kambadakone A, Thabet A, Gervais DA, Mueller PR, Arellano RS (2011) CT-guided celiac plexus neurolysis: a review of anatomy, indications, technique, and tips for successful treatment. Radiographics 31:1599–1621 10.1148/rg.316115526 21997984

[20] Tam A, Ahrar K (2007). Palliative interventions for pain in cancer patients. Semin Intervent Radiol.

[21] Raghavan K, Jeffrey RB, Patel BN, DiMaio MA, Willmann JK, Olcott EW (2015). MDCT diagnosis of perineural invasion involving the celiac plexus in intrahepatic cholangiocarcinoma: preliminary observations and clinical implications. AJR Am J Roentgenol.

[22] Zhang XM, Mitchell DG, Witkiewicz A, Verma S, Bergin D (2009) Extrapancreatic neural plexus invasion by pancreatic carcinoma: characteristics on magnetic resonance imaging. Abdom Imaging 34:634–641 10.1007/s00261-008-9440-4 18665418

[23] Ganten M, Krautter U, Hosch W (2007). Age related changes of human aortic distensibility: evaluation with ECG-gated CT. Eur Radiol.

